# Dynamic Impedance Model of the Skin-Electrode Interface for Transcutaneous Electrical Stimulation

**DOI:** 10.1371/journal.pone.0125609

**Published:** 2015-05-05

**Authors:** José Luis Vargas Luna, Matthias Krenn, Jorge Armando Cortés Ramírez, Winfried Mayr

**Affiliations:** 1 Health Technology Center, Reykjavik University / Landspitali—University Hospital, Reykjavik, Iceland; 2 Escuela de Ingeniería y Ciencias, Tecnológico de Monterrey, Monterrey, Nuevo León, Mexico; 3 Center of Medical Physics and Biomedical Engineering, Medical University of Vienna, Vienna, Austria; Duke University, UNITED STATES

## Abstract

Transcutaneous electrical stimulation can depolarize nerve or muscle cells applying impulses through electrodes attached on the skin. For these applications, the electrode-skin impedance is an important factor which influences effectiveness. Various models describe the interface using constant or current-depending resistive-capacitive equivalent circuit. Here, we develop a dynamic impedance model valid for a wide range stimulation intensities. The model considers electroporation and charge-dependent effects to describe the impedance variation, which allows to describe high-charge pulses. The parameters were adjusted based on rectangular, biphasic stimulation pulses generated by a stimulator, providing optionally current or voltage-controlled impulses, and applied through electrodes of different sizes. Both control methods deliver a different electrical field to the tissue, which is constant throughout the impulse duration for current-controlled mode or have a very current peak for voltage-controlled. The results show a predominant dependence in the current intensity in the case of both stimulation techniques that allows to keep a simple model. A verification simulation using the proposed dynamic model shows coefficient of determination of around 0.99 in both stimulation types. The presented method for fitting electrode-skin impedance can be simple extended to other stimulation waveforms and electrode configuration. Therefore, it can be embedded in optimization algorithms for designing electrical stimulation applications even for pulses with high charges and high current spikes.

## Introduction

Electrical stimulation is a powerful tool for diagnosis, treatment and function restoration in the human body. When applied transcutaneously, the impedance of the skin-electrode interface should be carefully considered, since previous reports have shown that it has a non-negligible direct influence on the stimulation outcome [1].

Many studies have reported spectroscopy measurements of tissue-electrode impedance, deriving mathematical models mainly on basis of electrical resistive-capacitive (RC) equivalent circuits or networks [2–5]. However, these spectroscopy models are based on recordings with sinusoidal low amplitudes currents, whereas clinical or research applications rely on impulse currents and much higher intensity levels. Typical electrical stimulation therapies use rectangular or triangular pulses at relatively low frequencies (<50Hz), and pulse widths of <2000μs for nerve and neuromuscular stimulation [6] or >30ms for denervated muscles [7]. Only few studies are found that focus directly on impedance dynamics under transcutaneous electrical stimulation and, most of them, only consider low-charge pulses [8,9]. It has been reported that current-voltage response on long pulses differ quite significantly from a behavior to be expected from conventional models based on fixed (within pulses) RC networks [10,11].

The better understanding of the skin-electrode interface impedance and the ability to predict its behavior under typical electrical stimulation conditions is important, since previous studies suggest that the impedance plays an important role for the performance and controllability of current-controlled (CC) and voltage-controlled (VC) stimulation [1]. Moreover, new approaches for improvements in selectivity of stimulation are based on novel shapes of stimulation waveforms. For example, it has been reported that the use of depolarizing pre-pulses can reduce fiber excitability [12], while hyperpolarizing pre-pulses can increase it [13]. Stimulus waveforms that include such pre-pulses work at low as well as at high intensities, therefore understanding the underlying mechanisms acting within the skin-electrode interface could be a key factor for development of new devices and stimulus designs.

Preliminary reports have showed that it is possible to develop a well correlating model of the skin-electrode interface impedance during application of typical transcutaneous electrical stimulation pulses [11] valid for both low and high impulse charges. The main aim of this work is to provide extended systematic experimental data on application of CC and VC biphasic rectangular stimuli via electrodes of various sizes and associated model calculations that allow to some extend general predictive calculations for other stimulus shapes and stimulation setups.

## Methodology

To understand observed impedance variations during transcutaneous electrical stimulation better, it is necessary to recognize influences of skin anatomy and electrical properties of its structures. Based on a literature study, a mathematical model that considers known mechanisms was developed that can be easily implemented in simulations. Further an experimental setup was defined to acquire data for calculating the variables of the model. Finally, the model is validated with simulation with different impulses shapes and the accuracy is discussed.

### Skin properties

The skin is a complex structure, but essentially composed by two layers: the epidermis (outer layer) and the dermis. Together they can vary in thickness depending on the body region, from 0.5mm at the eyelid up to more than 4mm at the foot sole.

The epidermis is composed principally of keratinocytes (~90%), melanocytes, Langerhans cells and Merkel cells [14]. It is, in general sense, a lipid-corneocyte matrix arranged in a flattened and irregular fashion [15], which is crossed by skin appendages (*e*.*g*. sweat glands and hair follicles) [16]. The outer layer of the epidermis is called *stratum corneum*. It is composed by a lipid lamellae-corneocyte matrix arranged in bilayers (between 25 and 100) and has an approximate thickness between 10 to 100μm [14,17,18].

The dermis is mainly composed by connective tissue (collagen and elastic fibers), but also fibroblast, macrophages and adipocytes [14]. It also has a great density of blood vessels [15], lymphatic vessels, sensory receptors, related nerves and glands [19], and follicles. The skin appendages density may vary upon the region of the skin. The sweat glands, for example, have a density between 1–6 pores/mm^2^ [14,15,20].

The dermis tissue, as well as deeper structures, has a stable environment that is rich in ions, which provides low and stable impedance levels. On the other hand, it is known that the *stratum corneum* acts as a barrier to hydrophilic and ionized-species movement and, consequently, represent the biggest resistive portion of the skin-electrode interface impedance. However, when wet, sweaty or bypassed, the conductance of the *stratum corneum* increases considerably [15].

It is documented that the body impedance during transcutaneous stimulation undergoes non-linear variations [19], which are mainly attributed to the skin-electrode interface. Some studies have localized this phenomenon in the *stratum corneum*, and showed a significant reduction of such variability, when this layer is removed [10,21,22].

**Fig 1:**
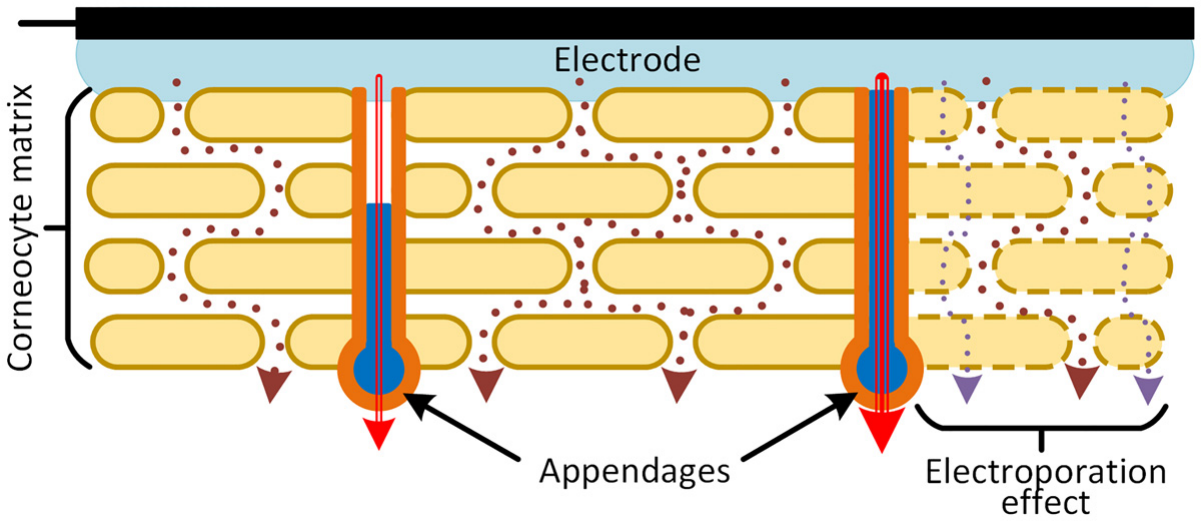
Simplify model of the skin-electrode interface, the stratum Corneum and its ionic pathways. Pathways in the appendages are shown in hard lines, and pathways going between the stratification of the *stratum corneum* matrix are shown in dotted lines. The electroporation effect on the corneocyte matrix is shown at the right, while the current flow increase due to the pore filling is shown in the appendages. Adapted from [2,5,11,20].

Once the electrode-electrolyte exchange occurs in the electrode (between the conductive material and an electrolyte solution, *e*.*g*. hydrogel), the ionic flow has at least two pathways into the *stratum corneum*: the first one that goes between the stratification of the corneocyte matrix layers; and a second one that goes through the appendages, mainly the sweat macropores (Fig 1). It has been shown that most of the ionic movement happens in the sweat pores, since the sweat is an electrolytic fluid and highly conductive [20].

#### Impedance variation mechanisms

There are many theories that try to explain the mechanisms that underlay the impedance variations on the skin. In a general sense, all of them describe structural changes that increase the ease of the electrical current to flow through the tissue, also known as conductivity (inverse of the resistance). The theories about how conductivity changes can be grouped according to the speed of the mechanisms that describe: instantaneous changes (within few microseconds [16,18,23]) and progressive changes (from milliseconds up to hours [2,10,23,24]).

The instantaneous changes are commonly addressed to the electroporation of the skin, which is the phenomenon where the membrane permeability to ions and macromolecules is increased by exposing it to a high electrical field (Fig 1) [25]. For transcutaneous electrical stimulation, the electroporation process occurs in both ionic pathways (matrix and pores), but at low amplitudes just the macropores are affected. This is because the lipid-corneocyte matrix is composed by multiple layers; therefore the electric potential drop in each layer is a small fraction of the applied voltage. On the other hand, macropores are composed only by few layers; therefore the potential drop across each layer is higher.

While instantaneous impedance variations have been widely investigated, there is still controversy about the mechanism of the slow changes. The most extended studies suggest that progressive increase of ions in the interface could be the answer [2,5,24]. Almasi & Schmitt concluded that impedance reduction in long-term applications is mainly related to the skin below the electrode getting increasingly hydrated or wet. This is due to the natural diffusion of ions and the expelled sweat that remains trapped below the electrode [24]. However these mechanisms work in the time scale of seconds up to hours and cannot explain the non-exponential behavior of the impedance within the duration of the electrical pulses. Grimnes and collaborators have proposed electro-osmosis and/or electrophoresis as mechanisms for ion movement, specifically for filling up the sweat conducts [2,5]. Both mechanisms are suitable to explain the impedance variations within the duration of the stimulus, since they start to work instantaneously and change along the pulse depending on the injected charge and speed of ions in the medium. The impedance variation due to the filling of sweat-pores (appendages) is represented in Fig 1, where bigger currents cross through the completely filled ducts rather than in the semi-filled ducts.

### Mathematical model

The developed mathematical model is based on two assumptions:

1. The electroporation effect is instantaneous, since the transient (few microseconds) cannot be detected at the sampling rate used.
2. The impedance of the deep-tissue and the bulk solution of the electrode remain constant for each subject.

**Fig 2:**
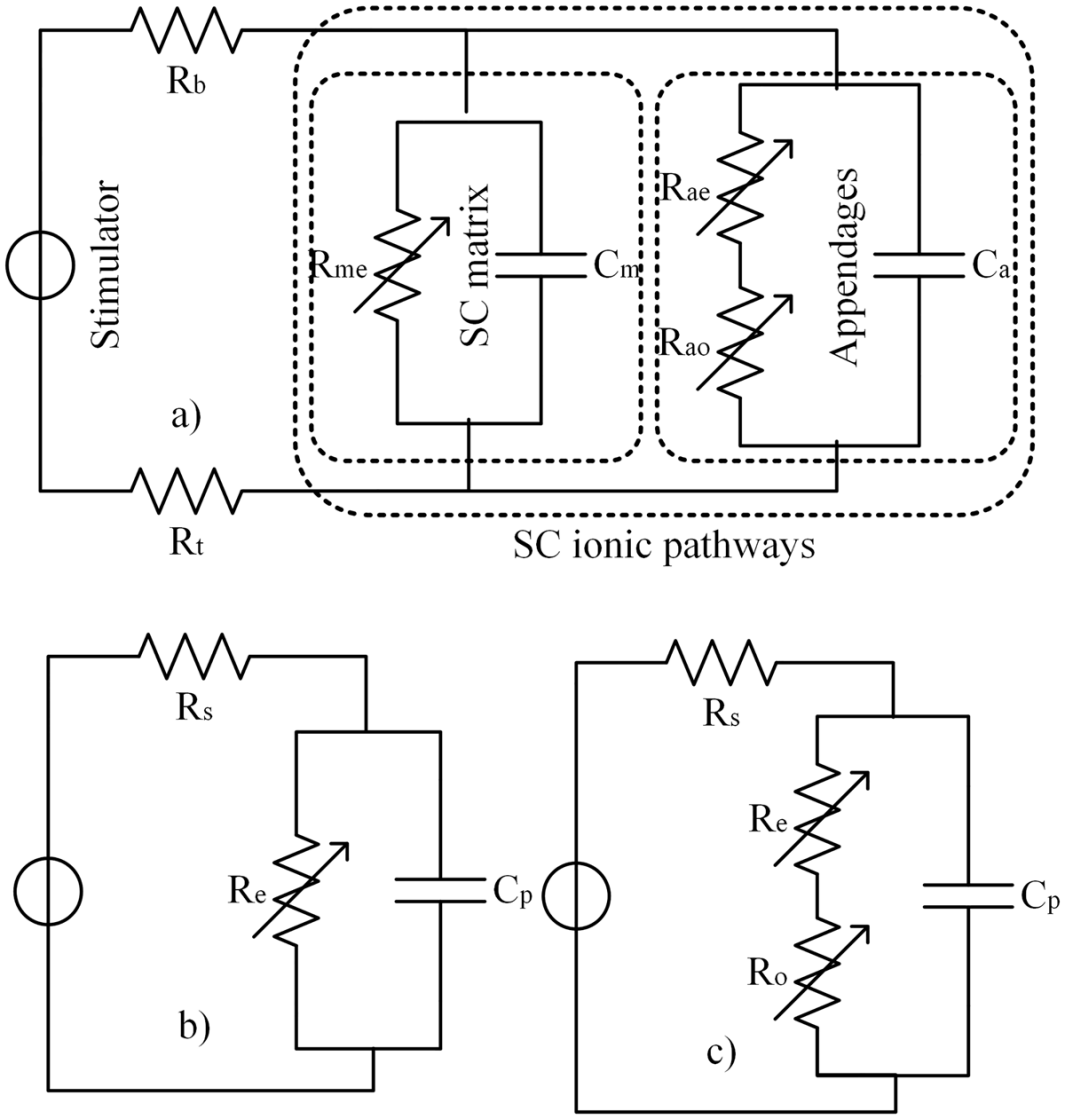
Skin-Electrode interface models. a) Full model considering electroporation and a charge-dependent effect; b) Simple model for low charge pulses; c) Proposed model.

Due to the complexity of the skin-electrode interface, impedance measurements cannot be done directly. Instead, an equivalent RC-network model can be used and tuned to describe the measured current-voltage response [19,26]. A widely use equivalent network is shown in Fig 2b, but many other models can be found in literature [2,3,9,10,19,21,27,28]. A modification of the model presented by Chizmadzhev *et al*. is shown in Fig 2a as a detailed model of the system [17]. In this full model, the bulk resistance of the electrolyte in the electrode (*R*_*b*_) and the tissue (*R*_*t*_) are presented separately. Also, both ionic pathways of the *stratum corneum* are considered with *R*_*me*_ and *C*_*m*_ for the lipid-corneocyte matrix, and *R*_*ae*_, *R*_*ao*_ and *C*_*a*_ for the skin appendages. Notice that the suffixes *e* and *o* stand for electroporation and charge-dependent (presumably electro-osmosis) effects respectively. Since the time constant for the lipid-corneocyte matrix is less than 1μs [17], the capacitor *C*_*m*_ is fully charged before the first sampling. Therefore *C*_*m*_ can be considered as an open-circuit and discarded from the model. Because *R*_*b*_ and *R*_*t*_ are constant and inherent to the system, they can be merged into a series resistance *R*_*s*_.

*R*_*ao*_ represents the resistance in the skin appendages when they are not completely filled with electrolyte. If the cases are considered, where the skin is fully wet or the charge-dependent effect is saturated (high pulse charges), then *R*_*ao*_ converges towards zero (*R*_*ao*_→0), and the resultant parallel resistance *R*_*e*_ (*R*_*me*_ || *R*_*ae*_) is defined by the impedance variations due to the electroporation of both ionic pathways (Fig 2b). Such simplification can be also applied to low charge impulses, where the charge-dependence becomes negligible and *R*_*ao*_ can be integrated as a constant in *R*_*e*_. In order to keep the duality for low and high charge pulses, the model shown in Fig 2c is proposed. Notice that the two resistances of the skin are placed in series instead of parallel (as should follow from Fig 2a), because the resulting values for a parallel model (*R*_*me*_ || *R*_*ae*_ and *R*_*ao*_) would lead to very high values. Otherwise, the proposed model shows similar values as to be found in literature.

The physiological phenomenon induced by electroporation is described as an increase of the tissue conductance *G*_*e*_. Preliminary data suggest that such changes can be modeled with a linear equation describing the course of *G*_*e*_ in dependence of the stimulation current as in Eq (1) [11]. This assumption leads to a *R*_*e*_ model similar to an initial exponential decay, which is consistent with other work to be found in literature [19].

$$\begin{array}{l} R_{e}=\frac{1}{G_{e}}\\ G_{e}(S)=a_{e}S(t)+b_{e} \end{array}$$ (1)

Where *S(t)* represents the stimulation current at a given time (*t*), *b*_*e*_ is the conductance at no excitation (*S* = 0mA), and *a*_*e*_ defines the conductance increase with respect to the current.

In contrast to the fast reacting electroporation, the electro-osmosis related flow rate into a semi-filled capillary is described as a function of the electro-kinetic potential and medium properties [5]. It is limited by the channel lumen and the repelling diffusion force generated by the electrolyte concentration increase near the electrode interface. In the final model, the charge-dependent effect (*R*_*o*_) goes from an initial value (given for typical skin conditions) down to zero, when the skin is completely humid.

$$R_{o}=\{\begin{array}{cc} R_{oi} & ,Ch<ACh_{th}\\ R_{oi}\left(1-\frac{1}{1+\frac{Aa_{o}}{Ch(t)-ACh_{th}}}\right) & ,Ch\geq ACh_{th} \end{array}$$ (2)

$$R_{oi}=R_{p}(t=0)-R_{e}$$ (3)

$$Ch(t)=\sum_{t=0}^{t}\left|i(t)\right|T_{s}$$ (4)

$$a_{o}=a_{a}I(t)+b_{a}$$ (5)

Where *R*_*oi*_ is the initial resistance that depends on the skin conditions, *A* is the electrode area in cm^2^, *Ch*_*th*_ is the charge threshold, where the electro-osmotic effect starts to be relevant (inflexion point) in [C/cm^2^], *Ch(t)* represents the charge of the pulse at time *t*, *T*_*s*_ is the sampling period and *a*_*o*_ is the variation of the electrical properties of the medium due to electroporation.

The equivalent RC model shown in Fig 2c follows, under typical conditions, the step response described by Eqs (6) and (7) for CC and VC respectively [29].

$$v(t)=i(t)R_{s}+i(t)R_{p}\left(1-e^{-\frac{t}{R_{p}C}}\right)$$ (6)

$$i(t)=\frac{v(t)}{R_{s}+R_{p}}-\left(\frac{v(t)}{R_{s}+R_{p}}-\frac{v(t)}{R_{s}}\right)e^{-\frac{t}{\left(R_{s}||R_{p}\right)C}}$$ (7)

$$R_{p}=R_{e}+R_{o}=\frac{1}{G_{p}}$$ (8)

$$G_{p}(S)=a_{p}S(t)+b_{p}$$ (9)

For both cases, the evaluation of the voltage-current response at t = 0 reflects the value of *R*_*s*_, because the capacitor acts as short-circuit. On the other hand, the final stable value corresponds to the relation of *R*_*s*_ and *R*_*p*_, because a fully-charged capacitor behaves as an open circuit. Notice also that if enough charge is applied (*e*.*g*. at the end of the pulse), then *R*_*o*_ converges to zero (*R*_*o*_→0) and *R*_*p*_ will depict the value of *R*_*e*_. *R*_*p*_ and *C* were estimated through a fitting based on Eqs (6) and (7) applied to the signal below *Ch*_*th*_. Similar to *R*_*e*_, *R*_*p*_ was modeled with a linear increasing of its conductance *G*_*p*_ as in Eq (9).

In order to model the dependence of *a*_*o*_ to the electroporation, stimulus amplitude was adjusted to different CC levels and then fitted with a linear regression based on Eq (5).

### Experimental Setup

A total of ten measurements sessions were considered. The protocol was approved by the Ethics in Research Committee and the Research Committee of the School of Medicine of the Tecnológico de Monterrey (Mexico), and conducted according to the principles of the Helsinki Declaration. A total of five neurological-intact volunteers participate in this experiment and all were informed of the experimental procedure, benefits and potential risks of the measurements, and signed an informed consent prior the measurements. The protocol included application of electrical stimulation on both legs of each volunteer. CC and VC stimulation were applied using commercial self-adhesive hydrogel electrodes (SN-50900, Hivox Biotek Inc., Taiwan) of two sizes: 5x10cm (ETD) and 5x5cm (ETC). For both CC and VC stimulation the stimulator output stage STMISOLA (Biopac Systems, Inc., USA) controlled via NI MyDAQ card (National Instruments, Inc., USA) was used. The electrical stimulation was applied to the anterior thigh (anodic electrode proximally) with an inter-electrode distance of 10cm. A custom code was implemented in LabView 2012 (National Instruments, Inc., Ireland) to control both the stimulator and the acquisition of the voltage-current response (sample rate 150kS/s).

The whole protocol was based on charge-balanced biphasic rectangular pulses and consisted of four stages: CC with the bigger electrodes (ETD), VC with ETD, CC with smaller electrodes (ETC) and, VC with ETC. The stimulation sweeps were done at 30,000μs per phase and varied from 1mA or 1V (in steps of 1mA or 1V) up to the pain threshold of the individual subject or the upper current limit of the stimulator (110mA).

### Data Processing

**Fig 3:**
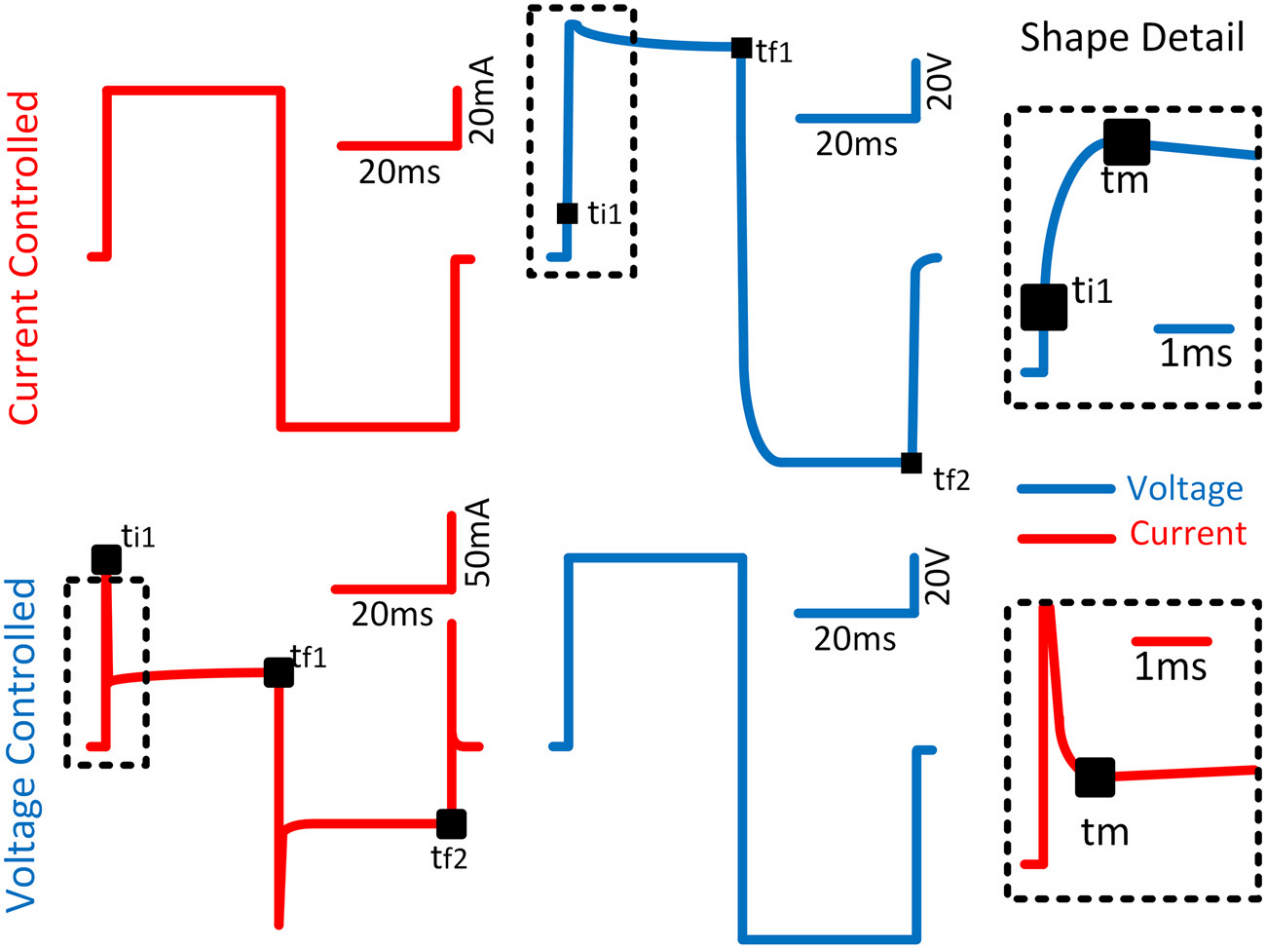
Representation of the current-voltage response during CC (top) and VC (down) with biphasic pulses of 30ms per phase. A detail of the first milliseconds is displayed for better appreciation of the inflexion point and the beginning of the pulse.

The data were post-processed in MATLAB (The MathWorks, Inc., USA). The acquired data were filtered with a moving average filter (span = 5) and an offset extraction on both signals was performed. Afterward, four time points were detected (Fig 3):

1. Beginning of the pulse (*t*_*i1*_): for CC, it is the time when the first current sample is equal or greater than the 99% of the pulse amplitude. For VC it is the time when the peak current sample appears with the leading impulse edge.
2. End of first phase (*t*_*f1*_): It is the time when first sample (after *t*_*i1*_) falls below 99% of the pulse amplitude in the first trailing edge.
3. End of second phase (*t*_*f2*_): It is the time when the last current sample (after *t*_*f1*_) remains below -99% of the pulse amplitude.
4. Inflexion point (*t*_*m*_): It is the time when the electro-osmotic effect starts modifying the exponential trace expected from RC circuit model calculations, which is defined as *Ch*_*th*_.

*R*_*s*_ and *R*_*e*_ were calculated from the apparent impedance at *t*_*i1*_ and *t*_*f2*_ (if stability is reached) respectively. The applied charge at the time points *t*_*m*_, *t*_*f1*_ and *t*_*f2*_ was calculated with Eq (4). Finally, *C* and *R*_*p*_ were estimated with Eqs (6) and (7) using the time segments where the applied charge was below *Ch*_*th*_. Time reference was the initiation of the leading impulse edge by the control input.

### Evaluation

In order to validate the model, a virtual bench was developed on Simulink (The MathWorks, Inc., USA). While the model was fitting mostly with the data from the first phase of the impulse, the simulation was done along the entire impulse. Then, the R^2^ and MSE values for the simulation were calculated.

## Results

The values of *R*_*s*_ remain quasi-stable along the whole stimulation amplitude variation range. A slight decreasing trend was observed in some cases, however, within practical stimulation intensity ranges, this decreasing can be considered as negligible. Table 1 presents the *R*_*s*_ values for each electrode size and each stimulus type (Mean±SD).

**Table 1:** Summary of estimated R_s_ and C values.

Variable	Stimulation Type	50cm^2^	25cm^2^
*R*_*s*_	CC	498±83Ω	670±99Ω
	VC	440±60Ω	596±86Ω
*C*	CC	194±52nF	106±25nF
	VC	176±62nF	83±31nF

Summary for all subjects of the *R*_*s*_ and *C* values based on the stimulation type and electrode size.

**Fig 4:**
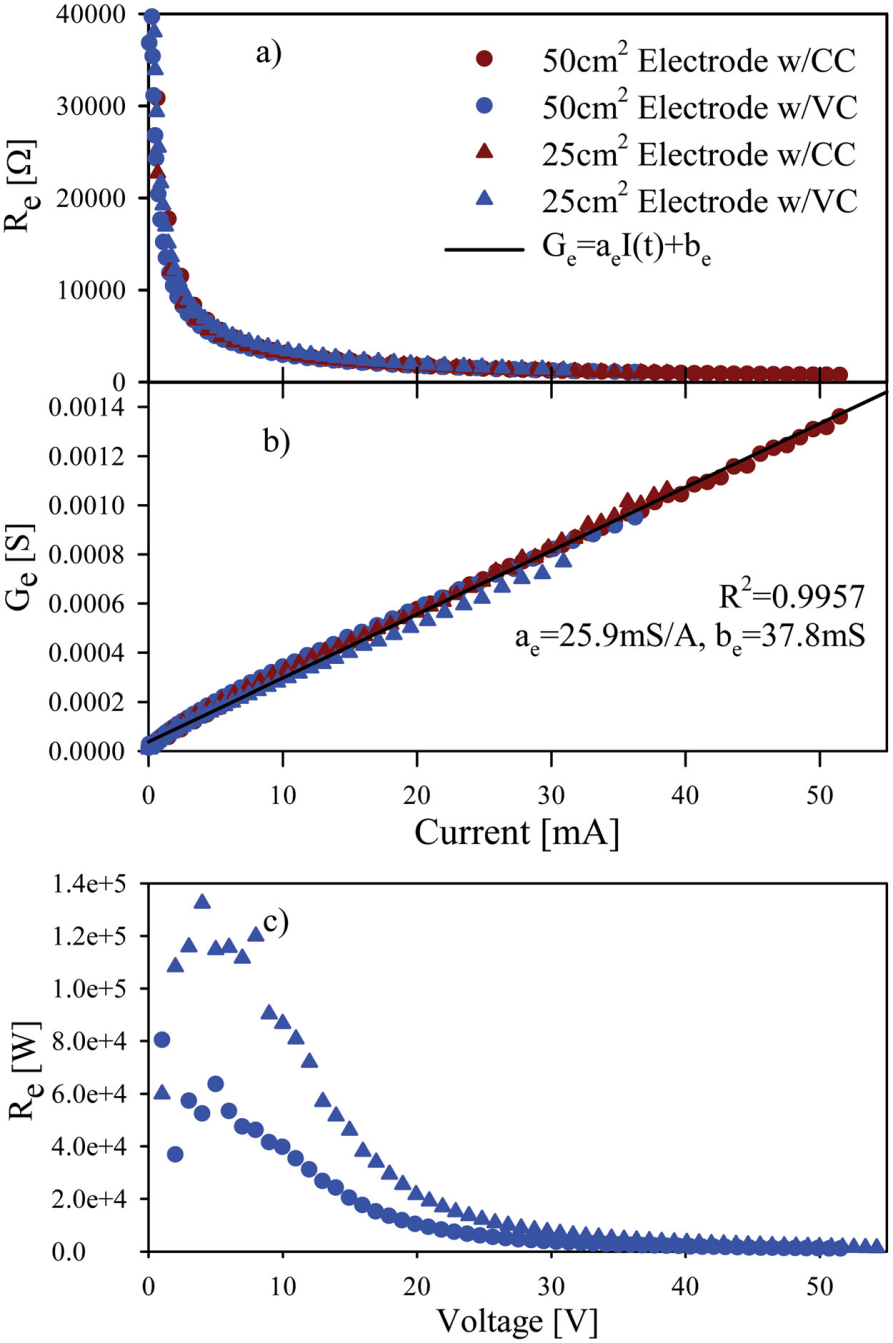
Typical estimated *R*_*e*_ values as function of a) current and c) voltage. b) *G*_*e*_ values as function of the current.

An exemplary result of *R*_*e*_ values (one subject) in relation to the current and voltage at *t*_*f2*_ is shown in Fig 4. The consistency of values in relation with the current, unlike the voltage, speaks for a current-dependence to the electroporation effect. Table 2 shows the average values of the coefficients for *G*_*e*_ estimation, and the R^2^ of the fitting.

**Table 2:** Summary of values to estimate G_e_ and G_p_.

Conductance	Coefficient	mean±SD	Range
	*a*_*e*_	24±4mS/A	18–30mS/A
*G*_*e*_	*b*_*e*_	64±30μS/A	14–104μS/A
	R^2^	0.979	0.956–0.997
	*a*_*p*_	14±3.5mS/A	7.6–19mS/A
*G*_*p*_	*b*_*p*_	56±33μS	7.8–109μS
	R^2^	0.939	0.866–0.982

Summary of the coefficients required to estimate *G*_*e*_ and *G*_*p*_ for all the subjects.

The inflexion point was easier to detect in VC pulses, where the charge injection is small and the charge-dependent effect appears progressively in the recorded traces. For CC impulses on the other hand, due to the fast charge injection, the detection of the inflexion point was not possible because only one measurement per subject (1mA with big electrodes) did not show the charge-dependent effect. The charge threshold average at small amplitudes was 350nC/cm^2^ and was similar for all subjects. High amplitude pulses were not considered since the huge current peak dynamic biased the detection to bigger values (~402nC/cm^2^).

**Fig 5:**
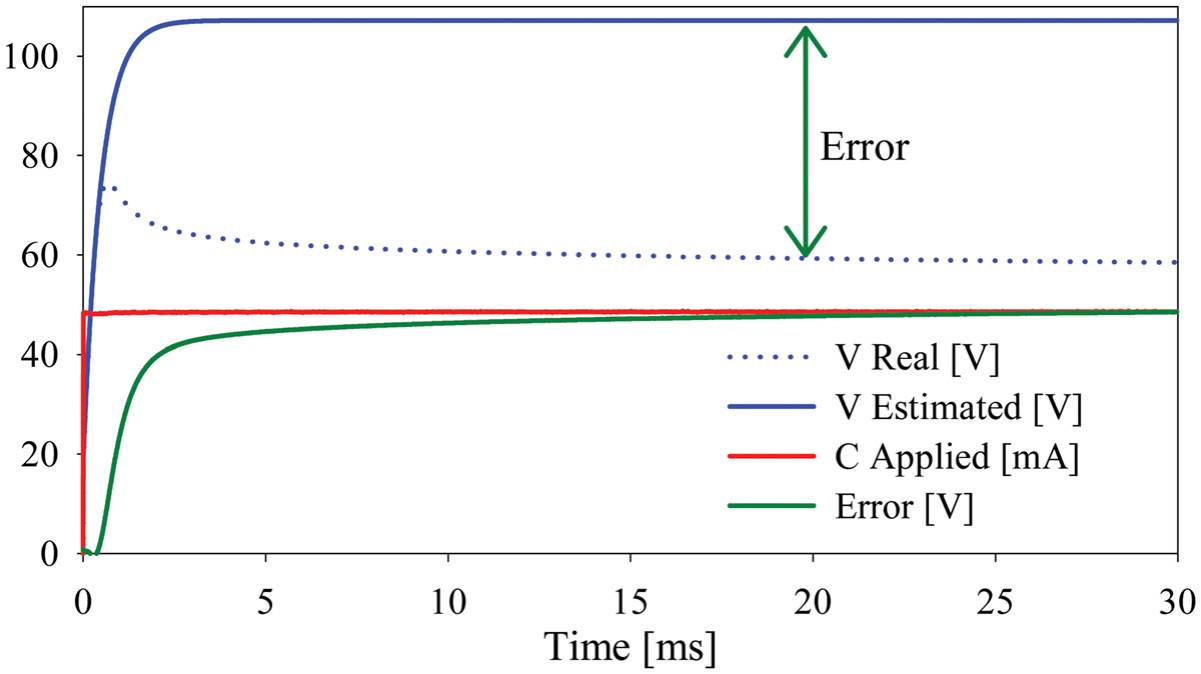
Typical voltage response estimation without considering the charge-dependent effect.

Once the beginning of the charge-dependent effect was defined, an estimation of the current-voltage response was done with the data preceding the *Ch*_*th*_ (Fig 5). Such estimation provides the *C* values shown in Table 1, as well as *R*_*p*_ and the error induced by the charge-dependent effect.

**Fig 6:**
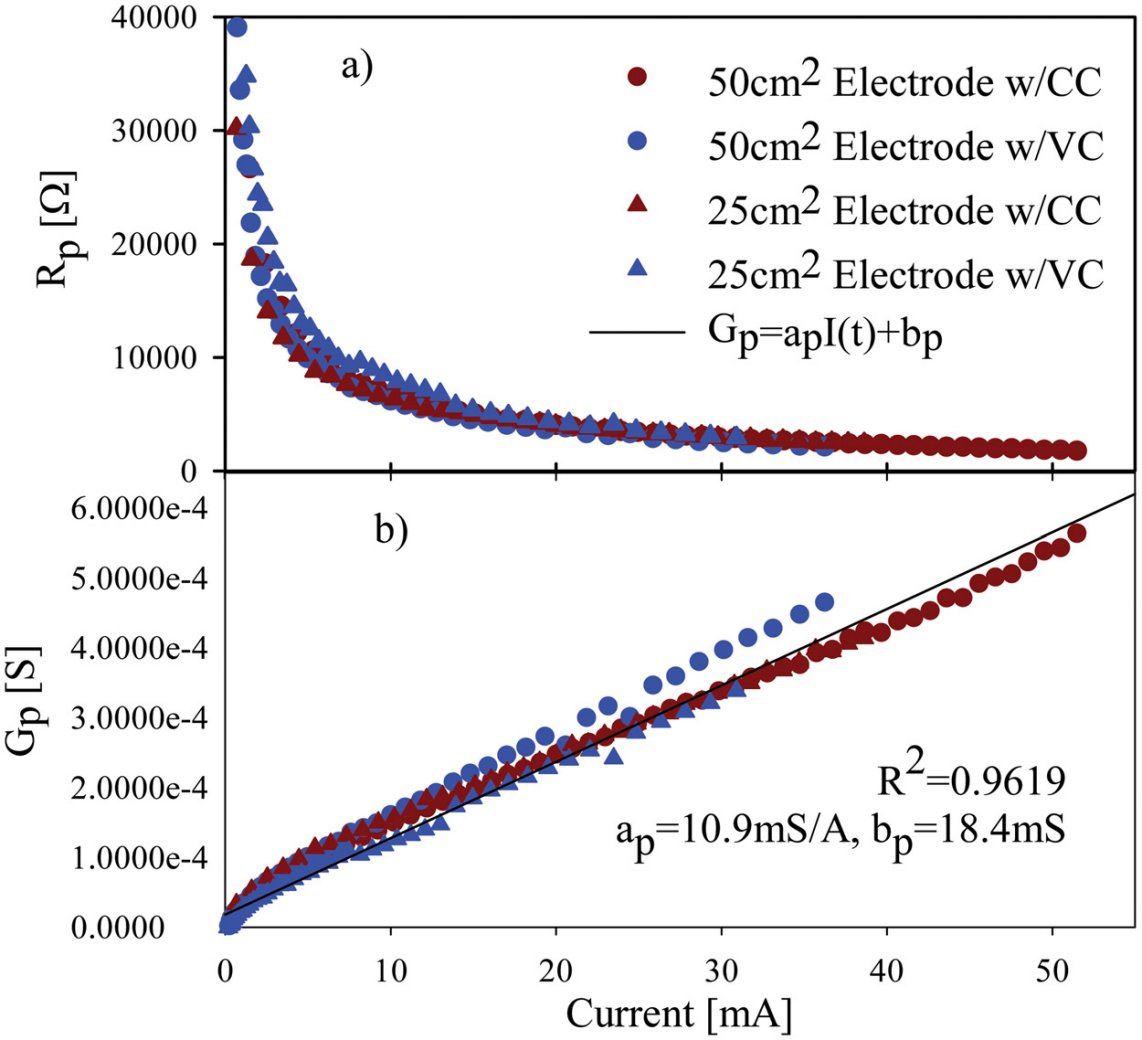
a) Typical estimated *R*_*p*_ values as function of current and; b) *G*_*p*_ values as function of the current.

*G*_*p*_ was modeled on basis of its linear dependence from current amplitude (Fig 6). It is important to notice that for VC stimulation the current at steady state was used as reference. The coefficients to estimate *G*_*p*_ are shown in Table 2. Finally, based on *G*_*e*_ and *G*_*p*_ estimation, *R*_*oi*_ was calculated based on Eq (3). The coefficient values for subject 9 (used example in all the figures) are summarized in Table 3.

**Table 3:** Coefficient values for Subject 9.

Symbol	Value	Units
*R*_*s*_ (50cm^2^)	CC:387,VC:356	Ω
*R*_*s*_ (25cm^2^)	CC:515,VC:481	Ω
*C* (50cm^2^)	CC:269,VC:259	nF
*C* (25cm^2^)	CC:139,VC:117	nF
*Ch*_*th*_	350	nC/cm^2^
*a*_*p*_	10.9	mS/A
*b*_*p*_	18.4	μS
*a*_*e*_	25.9	mS/A
*b*_*e*_	37.8	μS
*a*_*a*_	4000	nC/(A·cm^2^)
*b*_*a*_	70	nC/cm^2^

Summary of the required coefficients to implement the model specifically fitted for subject 9, which was used as an example in all the figures.

**Fig 7:**
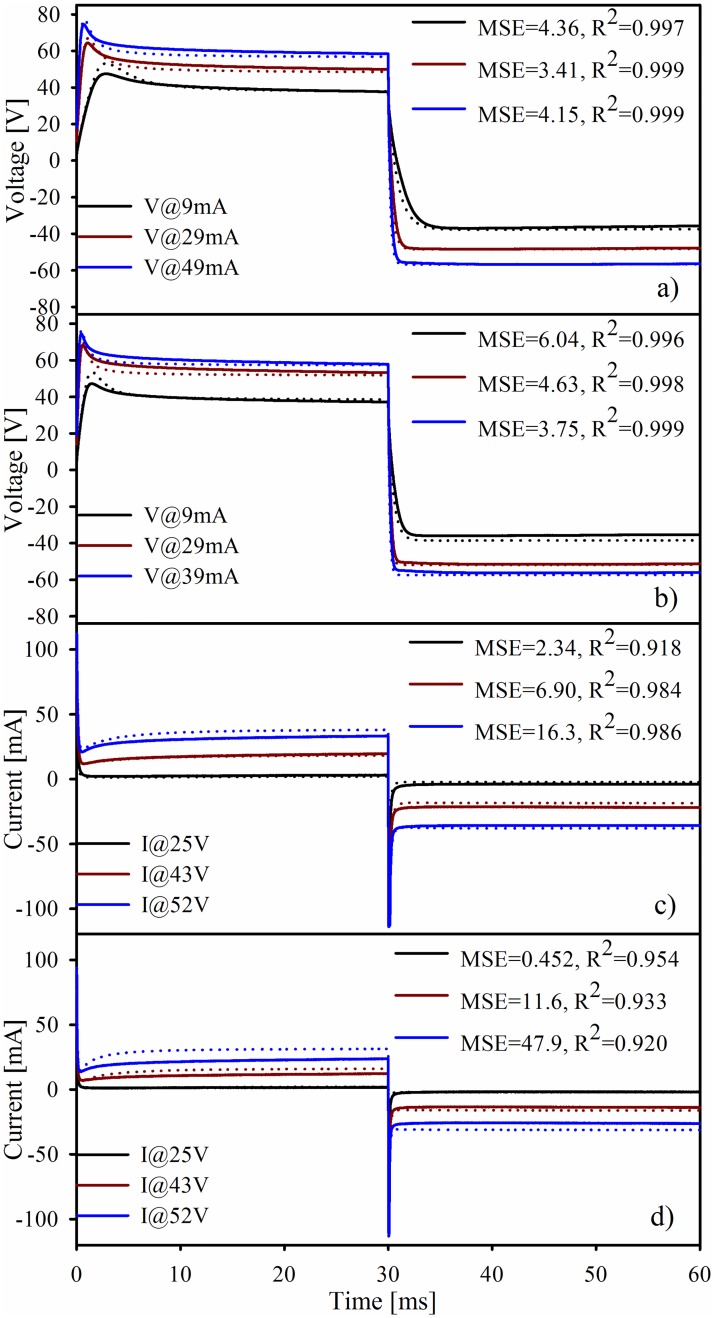
Comparison of exemplary measured values (hard line) and the simulation results (dotted lines) for a) CC with ETD, b) CC with ETC electrodes, c) VC with ETD and, d) VC with ETC.

Simulation was done for all pulses in CC and VC for both electrode sizes. Exemplary results are shown in Fig 7 along with the mean square error (MSE) and R^2^ of each impulse.

## Discussion

The slight decreasing trend of *R*_*s*_ values suggests that the electrical field induced in an organism leads to electroporation also in deeper lying tissue. As expected, the electroporation of deeper lying tissue is considerably smaller because of dispersion and attenuation of field strengths, but even more pronounced due to the low electrical resistance of the deeper tissue layers with smaller voltage drop in comparison to higher impedance layers at the surface (voltage divider effect). Additionally, differences between estimated values for CC and VC were noticed. It is possible that the measured differences are influenced by the assumption of an ideal pulse intensity step. For this reason, especially for CC mode, deviations in the initial resistance value could be originated by the capacitor charge at the moment of the first sample. However, the stability of the measurement is noticed in the consistent higher resistances values of the small electrodes, and a normal inter-individual variation range of resistance values.

The capacitance was modeled as a constant within all stimulation intensity ranges. As in *R*_*s*_, a slightly decreasing trend was observed in some cases. However, such decrease can be neglected since no relevant improvement was obtained in the accuracy of the final model when a variable capacitance was considered. In general, the capacitance of the big electrodes appeared as about twice the small ones, which directly correlates with the size relations and speaks for a proper estimation.

The estimation of *R*_*p*_ was done on bases of a current-dependence assumption. The linear model was fitting well, but like *R*_*e*_, the model cannot describe the entire control characteristic, since the value of the conductance (*G*_*p*_) must saturate in some point, which cannot be explored with the actual setup. Although the model lacks considering high intensity scenarios, it predicts essentially the entire clinically applicable amplitude ranges for intact skin sensation. Since the stimulation was performed below the pain threshold of the volunteers the outcome does not represent higher intensities as applicable in *e*.*g*. sensory complete spinal cord injured people.

The coefficient *a*_*o*_ was adjusted to give more weight to the beginning of each phase (and less to the saturating values towards the end). Since it is assumed that the charge-dependence is based on ion movement the electrical field, the current-dependence of *a*_*o*_ is also consistent with the change of the medium resistivity likely caused by the electroporation.

Although the model is based mainly on parameters fitted to the first phase, the dynamic simulation shows that it is able to predict the impedance behavior during both phases of the pulse with good correlation. It also is able to deal with different electrode sizes and stimulation control type. The R^2^ and MSE remain in acceptable levels in CC while in VC these parameters appear slightly degraded presumably because of small mismatches in the saturation levels.

Various mathematical models are presented in literature describing different applications and setups of electrical stimulation. However, most of them do not consider the variability of the skin impedance that, as shown in this work, may considerably change between pulses and within the pulse duration. The work presented here can be applied to larger electrical stimulation models either for pulse shape optimization or studying the viability of novel stimulation techniques. For example, Krouchev and colleges present a general mathematical model to design an energy-optimal electrical impulse for activating nerve fibers [30]. Based on this work, an extended model could be developed that includes transcutaneous electrode based applications, where the skin impedance plays an important role [1]. Danner and colleges developed a computer simulation to assess viability of transcutaneous spinal cord stimulation as an alternative to epidural stimulation [31]. In their work the skin impedance was considered constant, inclusion of the now presented interface model might lead to further refinement of the simulation, validity of interpretation and optimization of the stimulus parameters. The further achievement of the presented model could be in interpretation of the effects of subthreshold stimulus *e*.*g*. pre-conditioning pulses. It has been shown that pre-conditioning pulses may increase or decrease the fibers excitability [32], when applied transcutaneously such pre-pulses may substantially reduce the skin-electrode impedance. Therefore, the impedance reduction may play a secondary effect, which magnitude should be quantified.

## Conclusions

The proposed model is able to describe the skin-electrode interface impedance dynamically for different electrode sizes, with the same construction, and different stimulation modes (CC and VC). The mathematical model is based on the anatomical structure of the interface zone and its variables represent a biological phenomenon and structure.

The whole model is based on nine variables. With exception of *R*_*s*_ and *C*, all variables can be calculated from recordings along a single sweep of stimulation amplitudes and are valid for different electrode sizes and stimulation modes. *R*_*s*_ and *C*, on the other hand, must be calculated for each electrode configuration separately. It is important to notice that *G*_*e*_, *G*_*p*_ and *a*_*o*_ are modeled with a linear approach, therefore, only few points are required for its calculation, while *R*_*s*_, *C* and *Ch*_*th*_ require, essentially, only one reference point.

Unlike other works found in literature, this model is developed using typical stimulation parameters for clinical applications of transcutaneous electrical stimulation: pulse waveforms, stimulation amplitude ranges and stimulus duration. In the latter case, although the long durations are specifically necessary for activation of denervated muscles fibers, the impedance behavior is also directly relevant for shorter pulses, as applied for nerve stimulation, since it is identical in the initial phase of the impulse, independently from the further impulse duration.

The whole model is current-based, and it can be dynamically adjusted. This allows the predictive modelling of current-voltage responses for vastly any impulse and, therefore, the performance analysis and optimization of new pulse waveforms and stimulation techniques.
