# Correction: Dynamic Impedance Model of the Skin-Electrode Interface for Transcutaneous Electrical Stimulation

**DOI:** 10.1371/journal.pone.0130368

**Published:** 2015-06-05

**Authors:** José Luis Vargas Luna, Matthias Krenn, Jorge Armando Cortés Ramírez, Winfried Mayr

A funder is missing from the manuscript’s Funding section. The correct funding statement is as follows: JLVL was supported by the Mexican Council of Research and Technology (CONACYT) grant: 236850 (www.conacyt.mx). This work was also supported by the Vienna Science and Technology Fund (WWTF), Proj.Nr. LS11-057 (www.wwtf.at), and the Wings for Life Spinal Cord Research Foundation (WfL), Proj.Nr. WFL-AT-007/11 (http://www.wingsforlife.com). The funders had no role in study design, data collection and analysis, decision to publish, or preparation of the manuscript.
